# Quantitative relationship between volume of tumour cell units and their intravascular survival.

**DOI:** 10.1038/bjc.1978.33

**Published:** 1978-02

**Authors:** A. Lione, H. B. Bosmann

## Abstract

The derivation of the median volume (MV) and the geometric standard deviation (SDg) for a suspension of tumour cells quantifies the size and distribution of tumour cell aggregates in the suspension. Data collected in a group of 14 experiments shwoed a significant correlation of 0.80 (P less than 0.001) between the number of lung tumours formed by a suspension of B16 melanoma cells injected i.v. into C57BL/6J mice and the product of the MV and SDg of each cell suspension. These data define a size parameter of tumour cell suspensions that correlates with the intravascular survival properties of tumour cells.


*Br. J. Cancer* (1978) **37**, 248

### QUANTITATIVE RELATIONSHIP BETWEEN VOLUME OF TUMOUR CELL UNITS AND THEIR INTRAVASCULAR SURVIVAL

A. LIONE AND H. B. BOSMANN

*From the Department of Pharmacology and Toxicology, University of Rochester School of Medicine and Dentistry, and University of Rochester Cancer Center, Rochester, New York* 14642, *USA*

Received 16 July 1977 Accepted 23 September 1977

**Summary.—The derivation of the median volume (MV) and the geometric standard deviation ($SD_g$) for a suspension of tumour cells quantifies the size and distribution of tumour cell aggregates in the suspension. Data collected in a group of 14 experiments showed a significant correlation of 0·80 ($P<0·001$) between the number of lung tumours formed by a suspension of B16 melanoma cells injected i.v. into C57BL/6J mice and the product of the MV and $SD_g$ of each cell suspension. These data define a size parameter of tumour cell suspensions that correlates with the intravascular survival properties of tumour cells.**

THE formation of metastatic tumours in the lungs by blood-borne tumour cells can be studied using i.v. injected tumour cells. However, the elucidation of the roles of single tumour cells and cell aggregates in the formation of lung tumours has been hampered by the limitations of the existing methodologies for characterizing tumour cell suspensions. Experimental approaches which have been used to investigate this problem have necessitated the fractionation of tumour cell suspensions (Watanabe, 1954; Fidler, 1973; Thompson, 1974; Liotta, Kleinerman and Saidel, 1976), treatment with lectins (Ryd and Hagmar, 1977) or centrifugation (Hagmar and Norrby, 1973; Ryd and Hagmar, 1977) to prepare cell suspensions enriched in either single cells or aggregated cells. These manipulations may alter the surface properties and reproductive viability of the cell suspensions in ways that affect intravascular survival more than does the state of aggregation of the tumour cells.

This report describes an analytical method that is suitable to describe quantitatively the distribution of single cells and cell aggregates in tumour cell suspensions. The importance of tumour cell aggregates in the formation of lung tumours by i.v. injected B16 cells in C57BL/6J mice was investigated, using characterized cell suspensions in a series of 14 experiments.

### MATERIALS AND METHODS

*Cells.*—B16 melanoma cells were grown in Eagle's Minimum Essential Medium (MEM) containing 10% foetal calf serum, using Wheaton rotary flasks (0·5 rotations/min) and a 5% $CO_2$ atmosphere. Cells reached a density of approximately $4 \times 10^4/cm^2$ at confluency. The culture medium was routinely replaced with fresh medium 24 h before cells were used. Confluent cells were removed from glass with 0·025% trypsin in Tris-balanced buffered salt solution (8 min) and shaking. After centrifugation (650 *g*) the cells were suspended in Eagle's MEM (without serum) and maintained in suspension for 1 h by rotation in the cell culture apparatus at 3·0 rotations/min. This additional hour in suspension allows the tumour cell population time to establish a uniform shape and stabilize as a cell suspension after being removed from glass.

The cells were then again centrifuged, resuspended in 10 ml of Eagle's MEM without serum, and filtered through glass wool. The concentration of single cells and cell aggregates in the suspension was determined with an electronic particle counter (Coulter Counter model ZB; Coulter Electronics, Hialeah, Florida). The size distribution of the tumour cells was determined with an electronic sizing device (Coulter Channelyzer, Coulter Electronics). Cell viability, which ranged between 85 and 92%, was estimated with the trypan-blue exclusion test. Any tumour cell aggregate containing a cell that excluded trypan blue was categorized as a viable cell unit. The concentration of the cell suspension was adjusted to $10^5$ viable cell units (*i.e.* single cells and aggregates) per 0·2 ml of medium, and this amount was injected into the tail vein of C57BL/6J male mice aged 5–7 weeks. The viability and size distribution of the B16 cell suspensions did not change during the process of injection.

*Observation of tumour foci.*—After 10 days, the mice were weighed and killed. Lungs were removed, fixed in formalin-buffered saline for 12 h, and sequentially dehydrated with ethanol. The sequential dehydration of lung tissue in 50, 70, 80 and 95% ethanol–water solutions removed haemoglobin from the tissue and caused the lung tissue to become translucent. In 95% ethanol the translucent lung tissue provides maximal contrast for identification of melanin-containing tumour foci. The fixed, dehydrated lung tissue was next placed on the stage of a Bausch and Lomb dissecting microscope (Model 2-45L3). With backlighting, it was possible to detect and count tumour foci within the lung tissue as well as the more obvious superficial melanoma foci. To control for observer bias, all samples were randomly assigned a code number before lung tumours were counted. Repeated counts of lung tumours in selected samples showed the reproducibility in this counting system to be $\pm 6\%$.

Melanotic lung tumours were counted in the largest lobe of mouse lungs, the right dorsal lobe (labelled here "Lobe 5"). The correlation between the total number of lung tumours and the number of lung tumours in Lobe 5 was 0·96 ($P<0{\cdot}001$).

*Calculating the median volume and geometric standard deviation.*—The median volume and geometric standard deviation of each tumour cell suspension were calculated graphically (Kottler, 1950*a*, *b*; Smith and Jordan, 1964) using the frequency distribution data collected on a Coulter Channelyzer. Cumulative areas were measured with a Keuffel and Esser planimeter, Model 620005.

*Statistical analysis.*—The arithmetic mean (M) and the standard error of the mean (s.e.) were calculated for all data. Correlation coefficients were calculated using the assumption that the distribution of values analysed is a two-variable normal distribution (Dixon and Massey, 1969). To conclude whether a correlation coefficient ($r$) was significant, the probability that the value of $r$ is different from zero was determined using the $t$ statistic (Fisher, 1970). A $P$ value less than 0·05 was assumed to demonstrate statistical significance.

### RESULTS

Fig. 1 shows the size distribution of all particles in one of the 14 tumour cell inocula studied. The peak of the curve indicates that the most common-sized particle in this cell suspension, the single B16 melanoma cell, had a volume of $1{\cdot}41 \times 10^3\ \mu m^3$. The distribution of tumour cell aggregates present in the inoculum included aggregates larger than 4 times the volume of individual tumour cells.

The dotted line in Fig. 1 is an extrapolation of the major peak in the frequency distribution curve to zero. This extrapolation was made to eliminate the contribution of small cell debris and electronic noise from the area under the curve. Using the method described by Smith and Jordan (1964) and Kottler (1950*a*, *b*), the modified curve in Fig. 1 was converted to a linear form to facilitate further analysis of the data. The line shown in Fig. 2 was fitted to the points visually, with bias placed on a best fit with the points around the 50% region on the graph.

The volume of the 50% point—in this case $1{\cdot}82 \times 10^3\ \mu m^3$—is the median volume (MV) of the tumour cell suspension. This median volume represents the size of the particle which falls in the middle of the distribution shown in Fig. 1.

FIG. 1.—Frequency distribution of particle volumes in a suspension of B16 melanoma cells (see Table, Expt. 11). A Coulter Counter, model ZB, and a Coulter Channelyzer were used in data collection.

FIG. 2.—Log-probability plot of Fig. 1. $SD_g$ = geometric standard deviation; MV = median volume. "Percent" refers to the percentage of the total area under the curve in Fig. 1 falling between the plotted unit volume and the upper limit of the curve ($6{\cdot}52 \times 10^3 \mu m^3$).

The volume of particles that correspond to the 84·1 and 15·9% points in Fig. 2 are one standard deviation from the median volume of the plotted distribution. The ratio of the 84·1% volume to the 50% volume equals the ratio of the 50% volume to the 15·9% volume. This ratio is termed the geometric standard deviation ($SD_g$) and characterizes the scatter of particle volumes in the distribution around the median volume. The $SD_g$ of the frequency distribution shown in Fig. 1 is 1·71. The Table gives the data collected from 14 experiments in which the modal volume, the median volume, and the $SD_g$ of each tumour cell suspension were measured immediately before $10^5$ tumour cell units (single cells and aggregates) were injected into the tail vein of C57BL/6J mice. The survival of the tumour cells was measured by counting melanotic tumours in the largest lobe (Lobe 5) of the mouse lungs 10 days after the cells were injected. As shown in the Table, the median volume of the tumour cell suspensions ranged from

TABLE.—*The Viability, Modal Volume, Median Volume, Geometric Standard Deviation and Tumour Induction of* 14 *i.v. injected B*16 *Melanoma Cell Suspensions*

| Exp. No. | n† | Viability (%) | Modal vol. (×10³ μm³) | Median vol. (m.v.) (×10³ μm³) | $SD_g$‡ | MV×$SD_g$ (×10³ μm³) | Tumours in Lobe 5 (mean±s.e.) |
|---|---|---|---|---|---|---|---|
| 1 | 7 | 90 | 1·34 | 1·66 | 1·47 | 2·44 | 3±2 |
| 2 | 9 | 89 | 1·47 | 1·69 | 1·43 | 2·42 | 13±2 |
| 3 | 7 | 90 | 0·96 | 1·38 | 1·77 | 2·44 | 15±7 |
| 4 | 9 | 85 | 0·90 | 1·06 | 1·80 | 1·91 | 16±4 |
| 5 | 12 | 90 | 1·47 | 1·79 | 1·61 | 2·88 | 18±4 |
| 6 | 9 | 87 | 1·47 | 1·64 | 1·58 | 2·59 | 36±8 |
| 7 | 9 | 90 | 1·73 | 2·11 | 1·45 | 3·06 | 53±13 |
| 8 | 8 | 88 | 1·66 | 2·10 | 1·63 | 3·42 | 81±10 |
| 9 | 10 | 92 | 1·22 | 2·56 | 2·06 | 5·27 | 205±25 |
| 10 | 9 | 90 | 1·28 | 1·72 | 1·77 | 3·04 | 210±15 |
| 11 | 7 | 91 | 1·41 | 1·82 | 1·71 | 3·11 | 216±33 |
| 12 | 6 | 87 | 1·47 | 2·15 | 1·95 | 4·19 | 245±16 |
| 13 | 9 | 89 | 1·15 | 1·65 | 2·08 | 3·43 | 283±22 |
| 14 | 6 | 91 | 1·54 | 2·68 | 2·03 | 5·44 | 382±11 |
| r§= | | 0·33 | 0·09 | 0·62* | | 0·80** | |

* $P<0{\cdot}01$. ** $P<0{\cdot}001$.
† n = number of mice per group.
‡ $SD_g$ = geometric standard deviation.
§ r = correlation coefficient between each variable and number of tumours formed.

1·06 to 2·58 × 10³ μm³, and the values determined for $SD_g$ ranged from 1·43 to 2·08. The number of lung tumours produced by the i.v. injection of these cell suspensions ranged from 3 ± 2 to 382 ± 11.

For the data shown in the Table, the number of tumours produced by each cell suspension and the viability of each cell suspension do not correlate significantly ($r = 0{\cdot}33$; $P > 0{\cdot}05$). The modal volume and tumour formation also do not correlate significantly ($r = 0{\cdot}09$; $P > 0{\cdot}05$). However, the median volume of each cell sus-

FIG. 3.—The number of tumours found in Lobe 5 10 days after the injection of 10⁵ B16 melanoma cell units *v* the product of the median volume (MV) and geometric standard deviation ($SD_g$) of each cell suspension. Correlation coefficient = 0.80, $P<0{\cdot}001$.

pension and the number of tumours formed in Lobe 5 correlate at the 0·62 level ($P<0·01$).

Fig. 3 is a plot of MV $\times$ $SD_g$ and lung tumour formation for all 14 experiments. It shows that a significant positive correlation ($r = 0·80$; $P<0·001$) exists between the product of MV $\times$ $SD_g$ and the number of tumours formed.

### DISCUSSION

Liotta *et al.* (1976), Thompson (1974) and Fidler (1973) have all demonstrated the importance of cell aggregates in the formation of lung tumours by i.v. injected tumour cells. However, each of these studies compared lung tumour formation by one or more cell suspensions that were experimentally enriched in either single cells or aggregated cells. As pointed out by Ryd and Hagmar (1977), when the formation of lung tumours by two or more cell suspensions is compared, controlling for cell number injected is difficult yet essential. It is unclear how Thompson (1974) determined the total number of cells or cell aggregates when he compared lung tumour formation by large and small tumour cell aggregates. Although Thompson's data indicate that cell suspensions containing larger aggregates have a substantially higher colony-forming efficiency than suspensions of smaller aggregates, the injected number of large aggregates was nearly twice as large as the number of small aggregates in the reported data. Liotta *et al.* (1976) reported that neither single cells nor clumped cells produced more than 3 lung tumours per mouse, whether 0·5 or $1 \times 10^3$ tumour cells were injected. Fidler (1973) injected 10,000–12,000 clumped cells and 50,000 single cells into two groups of mice, in order to compare the number of lung tumours formed by each cell suspension. The mice that received the clumped cells developed more tumours. Ryd and Hagmar (1977) and Hagmar and Norrby (1973) reported no major differences in metastasis-yielding capacity between dispersed and aggregated cell suspensions. This observation may indicate that the use of lung weights and host survival time is not sensitive enough to quantitate the intravascular survival of tumour cells (Mellgren, 1976; Hagmar and Norrby, 1973; Ryd and Hagmar, 1977).

Based on the lung tumour formation of 14 characterized cell suspensions, the data reported here demonstrate that the number of lung tumours formed by the injection of $10^5$ B16 melanoma cell units (single cells plus aggregates) correlates significantly with the median volume and the product MV $\times$ $SD_g$ of the tumour cell suspensions.

The volumes of single cells and cell aggregates in the tumour cell suspensions studied here are log-normally distributed. The existence of a log-normal distribution is demonstrated graphically by the linear transformation that occurs when frequency distribution data are plotted on logarithmic probability axes (Smith and Jordan, 1964). As discussed in Kottler's monographs (1950*a*, *b*), the median volume and $SD_g$ are two quantitative terms that characterize a log-normal population.

The use of the median volume and $SD_g$ to characterize tumour cell suspensions not only introduces a new quantitative element into experimental work with i.v. injected tumour cells, but also provides a direct means of investigating the role of aggregated tumour cells in the formation of lung tumours. The extent of aggregation in a tumour cell suspension is reflected by the values of the median volume and $SD_g$. The value of $SD_g$ is derived from the slope of the log-normal plot of frequency distribution data. As scatter around the median volume of a cell suspension increases, the value of $SD_g$ increases. Since the distribution of particle volumes is not continuous on both sides of the median volume on a log-normal distribution (Kottler 1950*a*, *b*), a large value for $SD_g$ indicates the presence of more large aggregates in the cell suspension.

The product MV $\times$ $SD_g$ for each tumour cell suspension yields a single term which incorporates both the size and the distribution characteristics of each cell suspension. The median volumes of 14 cell suspensions and the numbers of lung tumours formed by those cell suspensions correlate significantly ($r = 0{\cdot}62$, $P < 0{\cdot}01$). The product $MV \times SD_g$ for the cell suspensions and the number of lung tumours formed have a larger positive correlation, equal to 0·80 ($P < 0{\cdot}001$). The positive correlations between these terms and lung tumour formation indicate that tumour cell suspensions with larger fractions of aggregated cells produce more lung tumours.

Aggregates of tumour cells could break up into smaller units during or after i.v. injection. Thus it may be suggested that the number of lung tumours formed when more aggregates were present in a cell suspension actually resulted from the injection of a larger number of tumour cells which formed from aggregates of cells. However, if cell aggregates did break up into smaller tumour cell units when injected, the relationship between $MV \times SD_g$ and the number of lung tumours formed would have an exponential form when plotted graphically. Our analysis has shown that the data in Fig. 3 corresponds best with a linear relationship between $MV \times SD_g$ and the number of lung tumours formed. Thus, we believe that cell aggregates are stable after i.v. injection.

Several factors may explain why aggregates of tumour cells survive better than single tumour cells in the blood stream. A group of tumour cells may establish a microenvironment which fosters tumour cell replication. Also, an aggregate of tumour cells cannot be eliminated by host reticuloendothelial defences as easily as a single cell. The surface properties of tumour cells that cause them to cohere while in suspension may also be involved in their adhesion to specific sites on the endothelial lining of blood vessels. The adhesion of tumour cell aggregates to select sites may also favour the growth and extension of intravascular tumour cells. In future work, the study of the plasma membrane properties of cell aggregates may reveal some of the critical factors that take part in the intravascular adhesion and survival of tumour cell aggregates.

Future work with tumour cell suspensions that have been quantitatively characterized may also prove useful for identifying therapeutic agents that can inhibit the survival of intravascular cell aggregates that form metastatic tumours in the lungs.

### REFERENCES

DIXON, H. J. & MASSEY, F. J., JR (1969) *Introduction to Statistical Analysis*, 3rd edn. New York: McGraw-Hill.

FIDLER, I. J. (1973) The Relationship of Embolic Homogeneity, Number, Size, and Viability to the Incidence of Experimental Metastasis. *Eur. J. Cancer*, **9**, 223.

FISHER, R. A. (1970) *Statistical Methods for Research Workers*, 14th edn. Edinburgh: Oliver & Boyd.

HAGMAR, B. & NORRBY, K. (1973) Influence of Cultivation, Trypsinization and Aggregation on the Transplantability of Melanoma B16 Cells. *Int. J. Cancer*, **11**, 663.

KOTTLER, F. (1950*a*) The Distribution of Particle Sizes. *J. Franklin Inst.*, **250**, 339.

KOTTLER, F. (1950*b*) The Distribution of Particle Sizes, Part II. *J. Franklin Inst.*, **250**, 419.

LIOTTA, L. A., KLEINERMAN, J. & SAIDEL, G. M. (1976) Significance of Hematogenous Tumor Cell Clumps in the Metastatic Process. *Cancer Res.*, **36**, 889.

MELLGREN, J. (1976) Quantitation of Metastases in Experimental Animals. In *Fundamental Aspects of Metastases*, Ed. L. Weiss. Amsterdam: North-Holland Publ. p. 243.

RYD, W. & HAGMAR, B. (1977) Effect of Cell Aggregation on Intravenous Tumor Transplantation. *Acta Path. Microbiol. Scand. Sec. A*, **85**, 405.

SMITH, J. E. & Jordan, M. L. (1964) Mathematical and Graphical Interpretation of the Log-Normal Law for Particle Size Distribution Analysis. *J. Colloid Sci.*, **19**, 549.

THOMPSON, S. C. (1974) The Colony Forming Efficiency of Single Cells and Cell Aggregates from a Spontaneous Mouse Mammary Tumour Using the Lung Colony Assay. *Br. J. Cancer*, **30**, 332.

WATANABE, S. (1954) The Metastasizability of Tumor Cells. *Cancer, N.Y.*, **7**, 215.


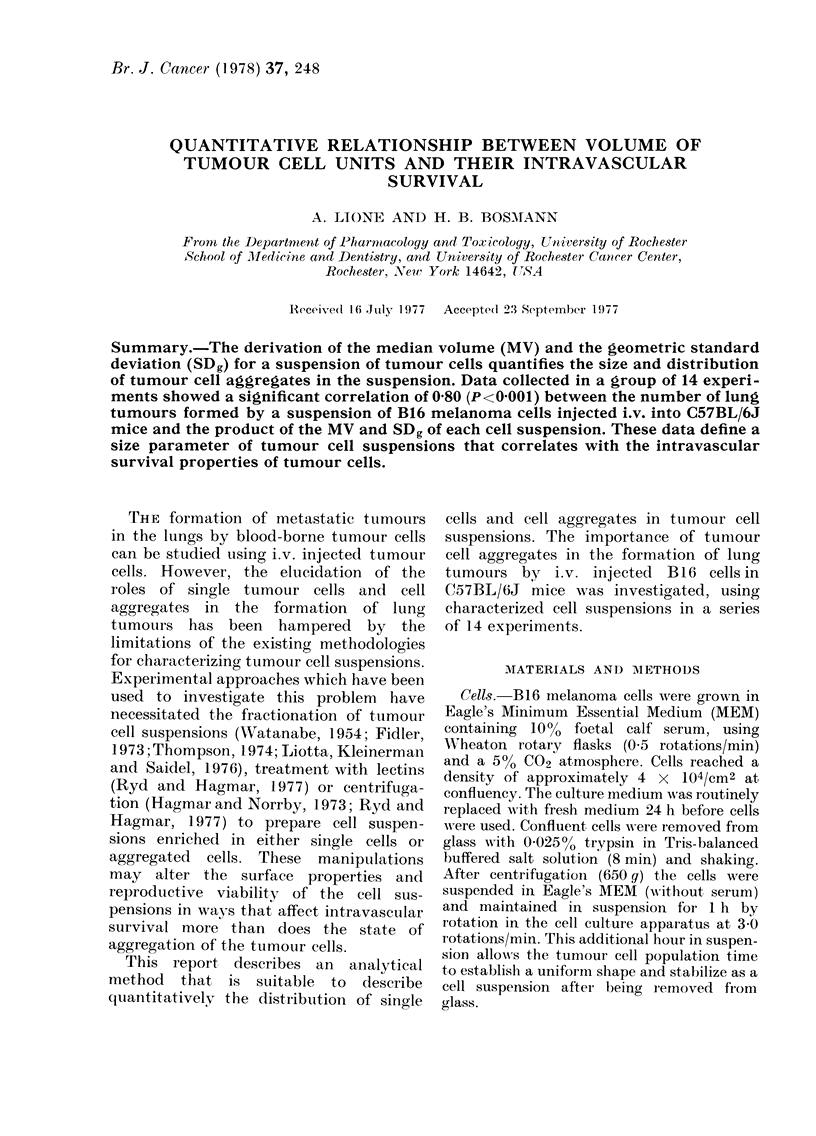

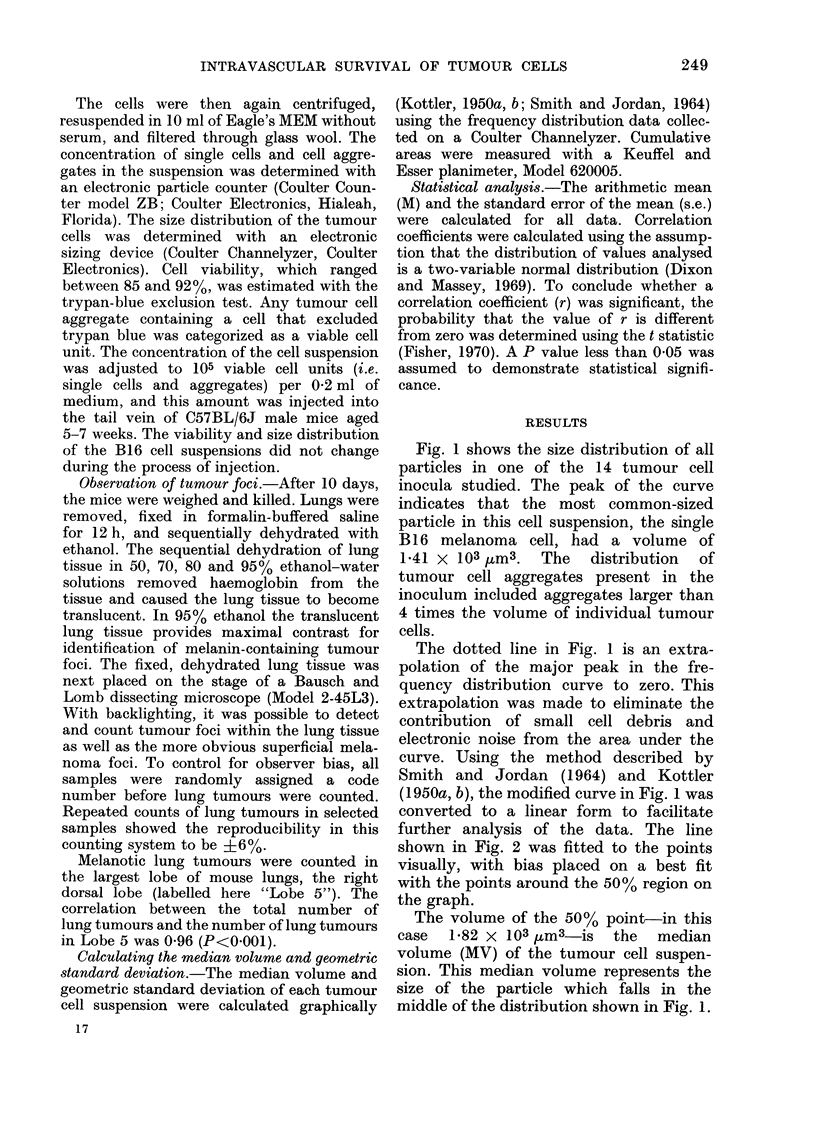

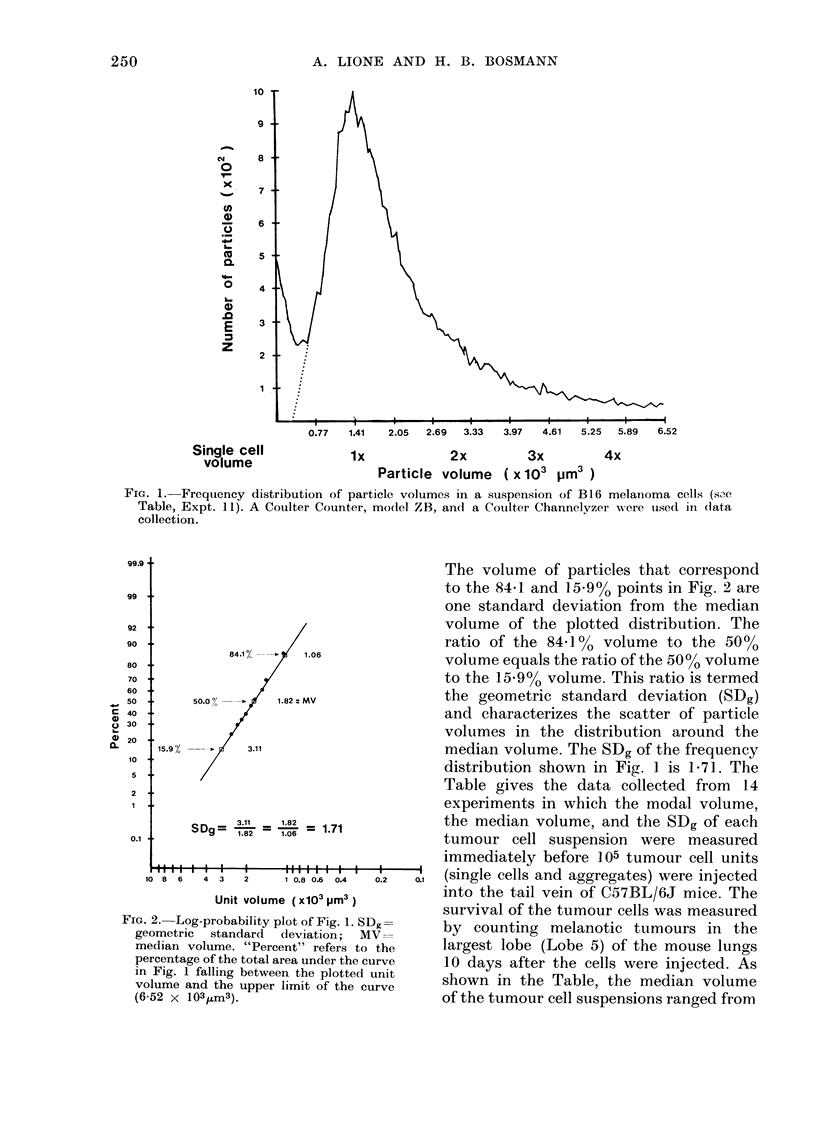

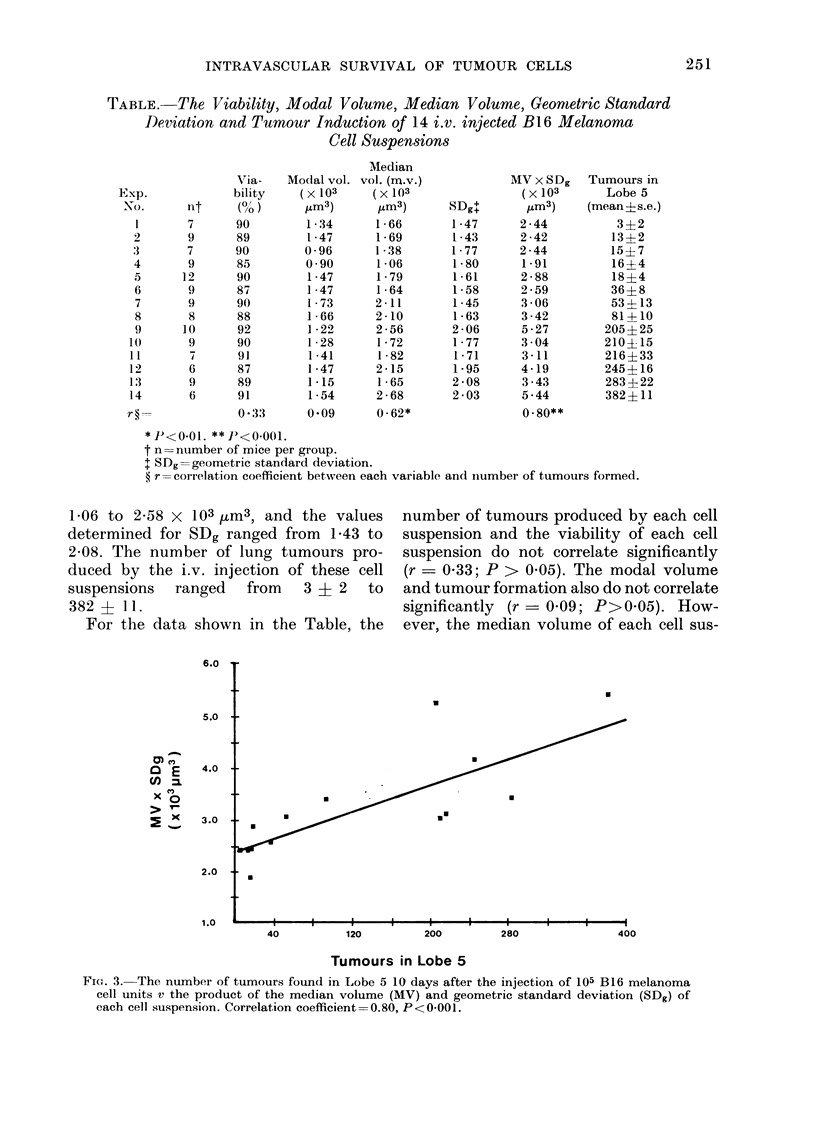

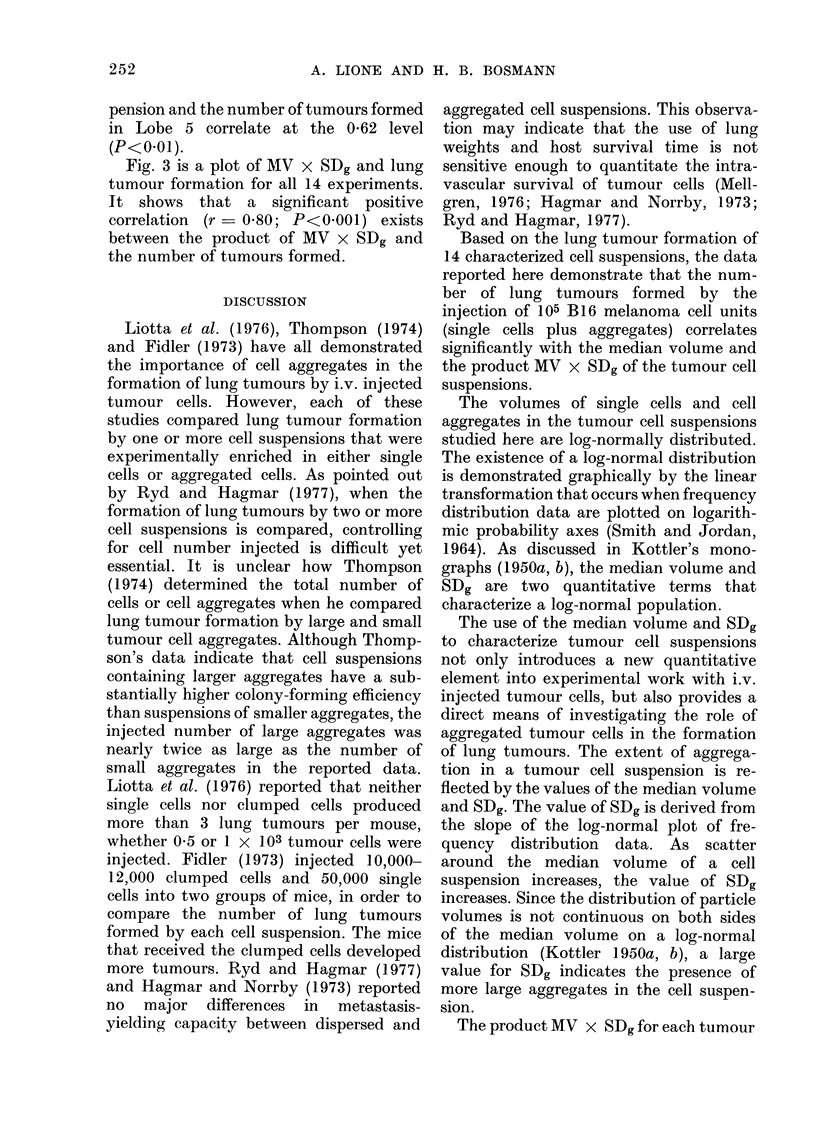

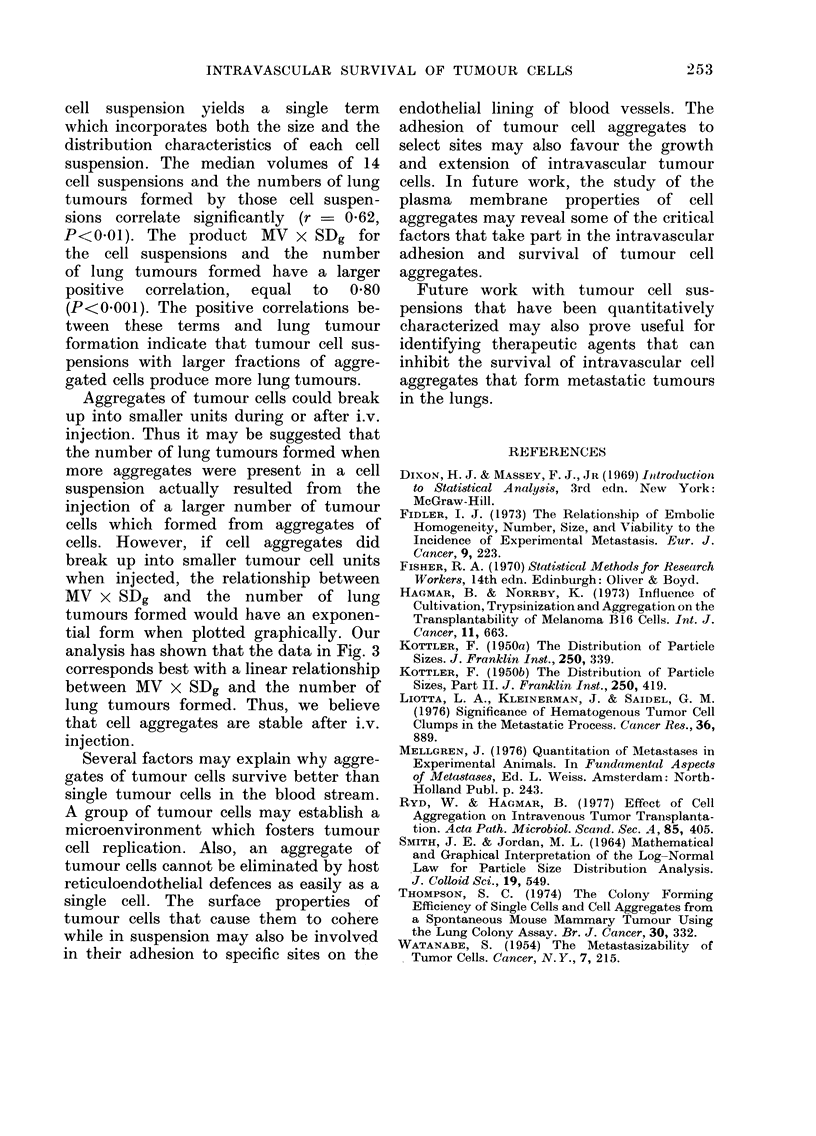


## References

[OCR_00769] Fidler I. J. (1973). The relationship of embolic homogeneity, number, size and viability to the incidence of experimental metastasis.. Eur J Cancer.

[OCR_00779] Hagmar B., Norrby K. (1973). Influence of cultivation, trypsinization and aggregation on the transplantability of melanoma B16 cells.. Int J Cancer.

[OCR_00793] Liotta L. A., Saidel M. G., Kleinerman J. (1976). The significance of hematogenous tumor cell clumps in the metastatic process.. Cancer Res.

[OCR_00805] Ryd W., Hagmar B. (1977). Effect of cell aggregation on intravenous tumor transplantation.. Acta Pathol Microbiol Scand A.

[OCR_00815] Thompson S. C. (1974). The colony forming efficiency of single cells and cell aggregates from a spontaneous mouse mammary tumour using the lung colony assay.. Br J Cancer.

[OCR_00821] WATANABE S. (1954). The metastasizability of tumor cells.. Cancer.

